# Occupational risks and musculoskeletal complaints among industrial workers: a cross-sectional study

**DOI:** 10.47626/1679-4435-2022-878

**Published:** 2023-08-08

**Authors:** Iraneide Nascimento dos Santos, Adriana Sarmento de Oliveira, Luciana Gonzalez Auad Viscardi, Jennifer Ariely Sales Suassuna, Amilton da Cruz Santos, Maria do Socorro Brasileiro-Santos

**Affiliations:** 1 Instituto Federal de Pernambuco, Ipojuca, PE, Brazil; 2 Undergraduate course in Fisioterapia, Universidade Anhembi Morumbi, São Paulo, SP, Brazil; 3 Laboratório de Estudos do Treinamento Físico Aplicado a Saúde, Departamento de Educação Física, Universidade Federal da Paraíba, PB, Brazil

**Keywords:** cumulative trauma disorders, musculoskeletal disorders, signs and symptoms, occupational risks, occupational health, transtornos traumáticos cumulativos, doenças musculoesqueléticas, sinais e sintomas, riscos ocupacionais, saúde do trabalhador

## Abstract

**Introduction:**

Work-related musculoskeletal disorders result from the overuse of the musculoskeletal system and insufficient time for the structures to recover. They are generally characterized by chronic pain, paresthesia, feeling of heaviness and fatigue, especially in the upper extremities, concomitantly or not, with an insidious onset.

**Objectives:**

To characterize musculoskeletal complaints and occupational risks in workers with work-related musculoskeletal disorders.

**Methods:**

A cross-sectional observational study of 60 participants in a Workers’ Health Reference Center with clinical and imaging diagnosis of work-related musculoskeletal disorders. The instrument used contained 30 questions about individual factors, occupational risks, and musculoskeletal abnormalities. The results were analyzed descriptively, and the chi-square test was used to assess associations with a significance level at p < 0.05. Data analysis was performed using BioEstat 5®.

**Results:**

Most participants were men (66.7%) working in the industrial sector. The most common complaint was pain (100%) in the shoulders (43.8%) and lumbar spine (22%), and the most common abnormalities were tendinopathies and intervertebral disc disorders. The following risk factors were identified: 8-hour workday (80%); repetitive gestures (86.7%); twisting (58.3%); bending (61.7%); standing (66.7%); manual work (96.7%); and 10-30 kg of weight handled (35%).

**Conclusions:**

A large number of workers exposed to biomechanical and organizational risks report musculoskeletal pain. Employers should check working conditions and adjust them, investing in health promotion and protection actions to effectively reduce the occurrence of these disorders.

## Abstract (pt)

**Introdução:**

Os distúrbios osteomusculares relacionados ao trabalho são decorrentes da utilização excessiva do sistema osteomuscular e da falta de tempo para recuperação dessas estruturas. Geralmente, são caracterizados por dor crônica, parestesia, sensação de peso e fadiga, sobretudo em membros superiores, concomitantes ou não, de aparecimento insidioso.

**Objetivos:**

Caracterizar as queixas osteomioarticulares e os riscos ocupacionais em trabalhadores portadores de distúrbios osteomusculares relacionados ao trabalho.

**Métodos:**

Estudo observacional com análise transversal, realizado com 60 participantes em um Centro de Referência em Saúde do Trabalhador, com diagnóstico clínico e de imagem dos distúrbios osteomusculares relacionados ao trabalho. O instrumento utilizado continha 30 questões sobre fatores individuais, riscos ocupacionais e alterações osteomusculares. Foram realizadas análises descritivas e, para avaliar as associações, foi utilizado o teste qui-quadrado, considerando nível de significância p < 0,05. As análises foram realizadas no programa BioEstat 5®.

**Resultados:**

A amostra foi majoritariamente masculina (66,7%) e do setor industrial. A queixa mais relatada foi a dor (100%) nos ombros (43,8%) e região lombar da coluna (22%), e as alterações mais encontradas foram tendinopatias e transtornos nos discos vertebrais. Alguns fatores de risco identificados foram: jornada de trabalho de 8 horas (80%); realizar gestos repetitivos (86,7%); torções (58,3%); deslocamentos (61,7%); adotar a posição bípede (ortostática) do corpo (66,7%); trabalho manual (96,7%); transporte de peso entre 10 e 30 kg (35%).

**Conclusões:**

Um grande número de trabalhadores expostos a riscos biomecânicos e organizacionais relata dores musculoesqueléticas. Cabe aos empresários verificar as condições de trabalho e ajustá-las, investindo em ações de promoção e proteção à saúde, para efetivamente reduzir a ocorrência desses distúrbios.

## INTRODUCTION

In recent decades, changes in the world of work have contributed to the emergence of new risks associated with work activities worldwide, with an increase in the rate of occupational diseases.^1^ In this respect, work-related musculoskeletal disorders (WMSDs) are one of the most common occupational diseases.^2,3^ WMSDs are a leading cause of absenteeism, disability, economic losses to society, and high treatment costs, being a major public health problem worldwide.^4,5^

WMSDs result from the overuse of the musculoskeletal system and insufficient time for the structures to recover. They are generally characterized by chronic pain, paresthesia, feeling of heaviness and fatigue, especially in the upper extremities, concomitantly or not, with an insidious onset.^6,7^ Complaints are most often the result of individual and occupational factors, such as workplace conditions and biomechanical and psychosocial factors.^6,8,9^ Biomechanical factors, such as inappropriate and repetitive postures and movements as well as static and dynamic loading conditions, and psychosocial factors related to work organization, such as increased overload and accelerated timeline,^1^ are occupational risks that cause a mismatch between the demands of the job and the physical abilities of the worker,^10^ lead to physical and emotional distress,^1^ and are major predictors of the onset of WMSDs.^8^ These risk factors, linked to the intensification of poor working conditions, affect a growing number of workers in the industrial and service sectors.^9^ In this scenario, it should be noted that musculoskeletal and connective tissue disorders, according to the International Classification of Diseases, were the second leading cause of leave from work, considering social security benefits such as disability or accident insurance, between 2012 and 2018.^11^ WMSDs are one of the most prevalent work-related chronic diseases in Brazil,^7^ with social, physical, and psychological repercussions,^12^ causing pain, suffering, absenteeism, disability, and reduced productivity.^5^

In view of the foregoing, it becomes relevant to conduct a study on the prevalence of WMSDs among workers to help those who deal directly with the problem to mitigate the social impact and damage that these disorders cause to individuals, corporations, public health systems, and social security programs. The objective of this study was to characterize musculoskeletal complaints and risk factors in workers treated in a specialized center.

## METHODS

An observational, analytical, cross-sectional study was conducted at the Workers’ Health Reference Center (Centro de Referência em Saúde do Trabalhador [CEREST]) located in the city of Cabo de Santo Agostinho, Pernambuco, Brazil. Currently, it is a regional center that provides technical support and assists in the processes of continuing education and training in occupational health. However, during the study period, it assisted workers from the city of Cabo de Santo Agostinho and surrounding areas who spontaneously came to the center or were referred by unions, municipal health facilities, and lawyers, among others. It is worth noting that CEREST is responsible for the municipalities within the area that is directly influenced by the Suape Industrial and Port Complex (Complexo Industrial e Portuário de Suape [CIPS]), considered one of the largest economic development projects in the country, a very diversified enterprise that, during the study period, attracted investors and generated income for workers in Pernambuco.

A non-probabilistic sample was used. The sample size was calculated by considering a finite population, based on the number of workers with WMSDs treated at CEREST in the previous year. From a population size (N) of 100 individuals, with a margin of error of 5% and a confidence level of 95%, 80 workers were initially included. However, 20 of these workers with a diagnosis withdrew participation or did not present a medical report confirming the diagnosis, resulting in a final sample of 60 participants.

Eligible participants were all workers, of either sex, performing any task, aged 18 to 59 years with a clinical and imaging diagnosis of WMSD based on medical reports. Workers with a history of joint, muscle, vascular, and rheumatic diseases, recent use of antibiotics, congenital malformations, or other musculoskeletal diseases were excluded. The study was approved by the Research Ethics Committee (CAAE: 42583320.1.0000.5208) and complied with the ethical aspects of research involving human subjects, according to Resolution No. 466/12 of the Brazilian Ministry of Health.

Participants were recruited among workers who spontaneously came to the center; in some cases, an appointment was scheduled. Workers with WMSDs were referred to the nursing office to be assisted by the center nurse who was also a researcher in this study. Workers who met the eligibility criteria were informed of the purposes of the study, and those who agreed to participate provided written informed consent. After obtaining consent, weight and height were measured in duplicate and an interview was conducted in an office at the center for data collection. After this step, the instruments were collected and stored for analysis.

A semi-structured questionnaire with 30 objective questions was used for the interview. It was adapted from the research guide for an ergonomic approach to musculoskeletal diseases of the Brazilian Ministry of Health,^6^ which includes a broad analysis of biomechanical, organizational, and psychosocial factors. The questionnaire has three domains with variables related to (1) individual factors, (2) occupational risks, and (3) musculoskeletal disorders. In addition to the questionnaire, a form developed by the authors was used to record the imaging findings. The independent variables were age and sex. Dependent variables included dimers, workload, body mass index (BMI), job position, complaints, overtime, repetitive movements, fine motor movements, heavy lifting, manual work, static posture, trunk twisting, trunk bending, neck flexion, two-finger pinch gestures, and monotony.

Data were tabulated in an Excel spreadsheet version 6.4, with independent double entry. After checking typing errors and inconsistencies, data analysis was performed using BioEstat 5®. For descriptive analysis, quantitative and qualitative variables were expressed as absolute and relative frequencies (%) and presented in graphs and tables. Mean, maximum, minimum and standard deviation values were calculated. The chi-square test was used to investigate associations of clinical complaints with sex and BMI, and body regions with BMI. A significance level at p < 0.05 and a 95%CI were considered.

## RESULTS

A total of 60 workers were evaluated, 66.7% male, with a mean age of 38.3 ± 9.2 years. The minimum and maximum ages were 23 and 59 years, respectively, distributed in the following ascending order: 23 to 30 years (23.3%); 31 to 40 years (38.3%); 41 to 50 years (26.7%); and 51 to 59 years (11.7%). Also, 27 workers were classified as overweight (45%), 26 as normal weight (43%), and 7 as obese (11%) (Table 1).

**Table 1:** Demographic data of workers with work-related musculoskeletal disorders

Variables	Workers’ profile
Age, years	38.3 ± 9.2
Sex, F/M [n (%)]	20/40 (33.3/66.7)
Weight (kg)	72.69 ±13.69
Height (cm)	1.68 ± 0.09
BMI (kg/m^2^)	
Normal weight	22.2 ± 1.54 (26/43%)
Overweight	27.4 ± 1.28 (27/45%)
Obesity	31.4 ± 1.54 (7/11%)

BMI = body mass index; F = female; M = male.

The main job positions held by most workers with WMSDs were machine operator (18.3%), production assistant (15%), which are essentially from the industrial sector, and general helper (10%). In addition, 61.7% of respondents reported having previously had a different job position, and 38.3% had never had a job position other than their current one. Regarding clinical complaints, 100% reported painful symptoms, 93.3%, reduced muscle strength, 65%, numbness, 58.3%, tingling, 45%, edema, 38.3%, cramps, 36.6%, shocks, and less than 10% each, tiredness, burning, dislocations, and throbbing.

Symptoms had been present for less than 5 years for 88.3% of workers. The body regions with the highest prevalence of musculoskeletal complaints were the shoulders (43.8%) and lumbar spine (22%). No worker reported complaints in the thoracic spine.

In the descriptive analysis of the association between sex and clinical complaints, there were no marked differences in relation to the reported complaints and pain, numbness, lack of strength, shocks, and tingling. Women more frequently reported symptoms of pain, cramps, and lack of strength. Complaints of throbbing, tiredness and dislocations were reported only by men (Table 2).

**Table 2:** Association between clinical complaints and sex in workers with work-related musculoskeletal disorders

Clinical complaints	Sex (%)	
	Male	Female
Pain	23.7	20.4
Numbness	15.9	12.2
Cramps	8.3	19.1
Lack of strength	20.7	19.3
Edema	6.5	16.3
Tingling	13.6	14.2
Shocks	8.8	8.1
Burning	0.6	1.1
Throbbing	0.6	-
Tiredness	0.6	-
Dislocations	0.6	-

Chi-square test p < 0.05.

According to the medical reports presented by the workers during the interview, the prevalent diagnosis was abnormalities in the shoulder region. However, the total number of diagnoses does not correspond to the total sample size, because some workers had more than one diagnostic hypothesis. Subscapularis, supraspinatus, infraspinatus, or calcareous tendinopathies and/or acromioclavicular osteoarthritis affected 39.1% of the participants, and 28.1% had intervertebral disc disorders, such as degeneration, herniation with and without nerve root compression, and osteoarthritis (Table 3).

**Table 3:** Distribution of the diagnoses of work-related musculoskeletal disorders

Clinical diagnosis	Frequency	
	Absolute	Relative (%)
Shoulder bursitis	4	6.2
Tendinopathy	25	39.1
Quervain tendinopathy	2	3.1
Lateral and/or medial epicondylitis	5	7.8
Carpal tunnel syndrome	5	7.8
Synovial cyst	1	1.6
Wrist synovitis and tenosynovitis	3	4.7
Spine abnormalities or osteoarthritis	18	28.1
Chondromalacia	1	1.6
Total	64	100

There was no association between symptoms and BMI in workers with WMSDs. However, regardless of BMI, the most common complaints were pain and reduced strength (Table 4). In addition, there was no association between BMI and musculoskeletal complaints, and most workers (91.7%) believed their body weight did not influence the development of WMSDs.

**Table 4:** Association of body mass index with clinical complaints and body regions in workers with work-related musculoskeletal disorders

Clinical complaints	BMI (%)		
	Normal weight	Overweight	Obesity
Pain	22.9*	23.5*	20.0*
Numbness	13.3	15.7	14.3
Cramps	6.7	6.9	14.3
Reduced muscle strength	20.9	21.7	14.3
Edema	12.4	8.7	11.4
Tingling	13.3	13.9	14.3
Shocks	8.6	7.8	8.6
Burning	-	0.8	2.8
Throbbing	0.9	-	-
Tiredness	0.9	-	-
Dislocations	09	-	-
Body regions
Right arm/elbow	11.4	6.7	14.2
Left arm/elbow	2.8	-	14.2
Right shoulder	20.0*	30.0*	28.6*
Left shoulder	20.0*	20.0	-
Right hand/wrist	17.1	6.7	-
Left hand/wrist	2.8	13.3	14.2
Cervical spine	2.8	-
Thoracic spine	-	-	-
Lumbar spine	20.0*	23.3	28.6*
Knee	2.8	-	-

BMI = body mass index.

*Chi-square test p < 0.05.

Of the evaluated workers, 88.3% were right-handed and 70% used both hands at work. There was an association of body regions and musculoskeletal complaints with the use of the right and left upper extremities. In addition, right-handers had a higher incidence of complaints in the right shoulder (26.5%), and left-handers, in the lumbar spine (37.5%) and in the right arm/elbow (25%). Regarding working years since the first job, the minimum was 3 years and the maximum was 49 years, with a mean of 19.2 ± 11.1 years. Regarding job tenure, the minimum length of time a worker had worked for their current employer was less than 1 year and the maximum length of time was 28 years, with a mean of 6.2 ±5.8 years. Regarding workdays, 80% of respondents had a daily workload of 8 hours, 8.3%, of 9 hours, and 6.7%, of 12 hours. As for overtime, most participants worked overtime (68.3%), and, of these, 30% worked between 5 and 10 extra hours, 23.3%, more than 10 extra hours, and 15%, less than 5 extra hours per week.

**Figure 1:**
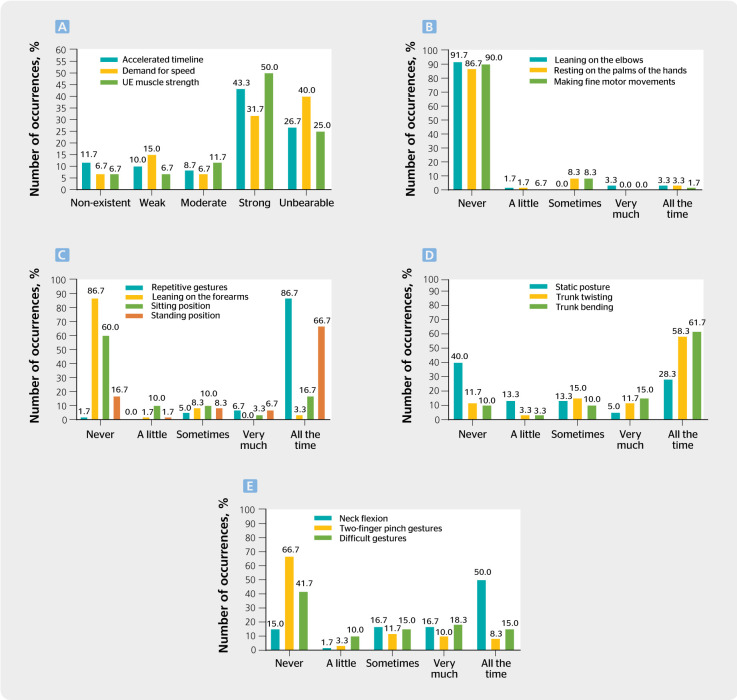
Number of occurrences of work activities, body postures and movements performed by workers. UE = upper extremities.

In the analysis of organizational factors, 26.7% of workers considered the accelerated timeline to perform their work activities to be unbearable. Regarding the demand for speed and intensity of muscle strength required from the upper extremities, 40% of workers classified them as unbearable and 50%, as strong, respectively (Figure 1, panel A). Regarding body posture during work activities, 91.7% of respondents reported never working leaning on their elbows, 86.7%, never resting on the palms of their hands, and 90%, never making fine motor movements (Figure 1 , panel B). Also, 86.7% and 66.7% of workers made repetitive gestures and worked in a standing position all the time, respectively. However, 86.7% and 60% of workers never leaned on their forearms or worked in a sitting position, respectively (Figure 1, panel C). In addition, 40% of workers never worked in a static posture, whereas 28.3% maintained a static posture all the time, 58.3% twisted the trunk and 61.7% bent the trunk all the time (Figure 1, panel D). Regarding physical work overload, 96.7% of respondents performed manual work and 76.7% handled heavy equipment during their work activities – of these, 13.3% up to 10 kg, 35% between 10 and 30 kg, 21.7% between 30 and 60 kg, and 6.7% more than 60 kg.

As for neck flexion, 50% of workers performed it all the time, 66.7% never adopted two-finger pinch gestures, and 41.7% never made difficult gestures (Figure 1, panel E). Regarding perceived physical exertion at work, 65% of respondents considered themselves strong enough to perform their work, 95.5% felt tired during working hours, 43.3% believed they were more tired at the end of the workday, and 38.3%, in the middle of the workday. As for the monotony of the task, 68.3% considered the work very monotonous, and about work organization, 70% of respondents reported not having rest breaks, and among those with a scheduled rest break, 13.3% had 10- to 20-minute breaks, whereas the remaining responses had percentages less than 10%.

Regarding extra-work activities, 25% of workers responded that they did household chores, 5% sewed, and 1.7% played a musical instrument. However, most workers (68.3%) did not perform extra-work activities. It should be noted that none of the workers engaged in physical activity.

## DISCUSSION

The present study, which aimed to characterize musculoskeletal complaints and occupational risks in WMSDs, identified that the sample: (a) consisted mostly of young adult male workers from the industrial sector; (b) reported 100% of painful complaints, with a higher prevalence of symptoms in the shoulders and lumbar spine, with tendinopathies and intervertebral disc disorders as the most common abnormalities; and (c) performed their work activities for less than 5 years at their current company, with an 8-hour workday and weekly overtime, adopting unnatural postures such as repetitive gestures, trunk twisting and bending, and standing position, with manual work and 10-30 kg of weight handled. As in previous studies,^13,14^ most workers were 31 to 40 years old (38.3%), considered the productive years of life, which can significantly influence their work capacity and, depending on the risk factors, contribute considerably to the progressive development and maintenance of the disorders.^12^ Conversely, the likelihood of developing WMSD-related symptoms increases with advancing age, since, during the aging process, there is progressive degradation of brain and musculoskeletal functions, negatively affecting muscle strength and flexibility.^15^ It is worth mentioning that degenerative tendon injuries can develop in adults above the age of 35 years, when tendon tissue repair is no longer as effective as before, both in terms of speed and quality.^10^

Most participants were men (66.7%). The higher occurrence of WMSDs in men may be due to the main job positions observed in this study, with a higher prevalence of machine operators (18.3%) and production assistants (15%), industrial activities that generally employ male workers. It should be noted that these activities require muscle strength, especially in the upper extremities and spine, which may have contributed to the onset of WMSDs.

In this study, in addition to the lack of identification of BMI as a risk factor for WMSDs, most workers also reported that they did not think their body weight influenced the development and/or maintenance of these disorders. However, it is important that workers become aware of the impact of overweight on musculoskeletal structures,^16^ which can impair functional performance, as indicated by higher rates of strength loss, declines in task performance, and increases in discomfort, consequently influencing the postures adopted and making work more demanding.^17^

Another factor to be discussed is the adoption of unnatural postures outside the workplace. When asked about activities performed outside the workplace, most workers with WMSDs responded that they did not perform extra-work activities. However, 25% of them did household chores, which may have contributed to the development of these disorders if performed improperly. Regarding job tenure, it was not possible to determine whether the WMSDs were a result of job tenure because most participants had worked for their current employer for less than 5 years, and 41.7% had a total length of working time since the first job of 10 to 19 years. Regarding organizational risk factors, the most common factors were accelerated timeline to perform work activities and an 8-hour workday plus overtime, mostly between 5 and 10 extra hours per week. However, the study by Oranye & Bennett^3^ pointed out that the risk for WMSDs is more closely related to the physical tasks than to the amount of time spent on such tasks.

In the analysis of repetitive gestures, most workers performed them all the time during the workday, found the work activity monotonous, in addition to considering both the demand for speed in performing their activities and muscle strength required from the upper extremities to be unbearable. These factors altogether add an overload to the work tasks, which is incompatible with human physical and psychological capacity. Accordingly, studies have shown that workers with WMSDs report work overload, pressure to produce, lack of control over the pace of work,^2^ no rest breaks, excessive overtime, and monotonous and repetitive tasks.^9^

Regarding body posture during work activities, this study pointed out that most workers performed their activities in a standing position all the time. This position is five times more likely to produce pain in more than one body region,^18^ in addition to posing biomechanical risks. A static posture requires a low level of muscle strength and favors the maintenance of postural misalignment for prolonged periods.^10^ Furthermore, biomechanical risks, such as inappropriate sitting/standing posture, hand position during work, and repetitive movements, and psychosocial risks may lead to the onset of musculoskeletal disorders in workers.^2^ Likewise, prolonged standing and sustained postures, particularly those associated with twisting and bending the trunk forward, are considered risk factors for the development of musculoskeletal disorders,^10^ which can affect different parts of the body.

In this study, workers complained of pain mainly in the shoulders and lumbar spine, body regions that are most frequently affected by WMSDs. The most prevalent diagnoses were subscapularis, supraspinatus, infraspinatus, or calcareous tendinopathies and/or acromioclavicular osteoarthritis, followed by intervertebral disc disorders. These disorders probably occurred due to prolonged standing and trunk twisting and bending, since the execution of dynamic and repetitive movements involving the spine, mainly lateral flexion and rotation, may increase body overload and are highly harmful.^10^

Regarding the use of the upper extremities in work activities, nearly 100% of the workers who performed manual work were right-handed, and most used one hand more than the other, more specifically, the right hand. This finding was associated with the body region with the highest incidence of WMSDs, the right shoulder. In addition, the biomechanics adopted at the workplace may pose a moderate risk to the onset of chronic musculoskeletal pain both in the upper extremities and in other regions, such as the lumbar spine.^14^

Regarding work capacity, most respondents considered themselves strong enough for their assigned tasks and rated their activities as difficult to perform. However, the data on musculoskeletal complaints were not compatible with this information. It should be noted that current job positions often require different physical responses from workers in order to get the assigned tasks accomplished, which are not found in all people. Therefore, there is a need to adjust the working conditions and workstations^19^ according to ergonomic recommendations.^10^

Another risk factor for WMSDs is heavy lifting. In this study, most respondents reported that their job required lifting and handling 10 to 30 kg of equipment. Depending on the weight of the equipment and how the worker handles it, there may be additional stress on body structures, particularly the spine.^10^ There is already a natural effort of the spine to maintain an upright posture, thus rendering it more sensitive to external forces acting on the body.^20^ Therefore, workers should be informed of how to handle and how much weight they should handle during their work activities, as well as of the potential musculoskeletal damage that may occur if they do not comply with these rules.^21^ It is worth noting that Law No. 6,514, of December 22, 1977, in its Articles 198 and sole paragraph, which provides for the Prevention of Fatigue, specifically on the maximum weight that an employee should be allowed to handle individually, determines a maximum weight of 60 kg for men, except for special provisions relating to the work of minors and women.^22^ Although a law exists, it does not consider each individual’s work capacity or their different physical conditions and particular resistance to physical exertion. As a result, workers continue to get ill, highlighting the multifactorial nature of WMSDs.^6^

Musculoskeletal disorders can negatively affect the work routine, and our sample confirmed the need to change body position during work due to pain in the neck, shoulders and/or arms, back, or legs. Workers should have a rest break at least once a day, or even several breaks a day, due to pain symptoms. In addition, most workers reported feeling tired during working hours, especially at the end of the workday. Based on this information, it can be concluded that it is essential to have rest breaks during work,^2^ even if there is no consensus on the number of breaks required for the different workloads and professions. In general, frequent short rest breaks are preferred. In electronic processing activities, workers should be given at least a 10-minute rest break every 50 minutes.^21^ Furthermore, a workplace exercise program is recommended and workers should be encouraged to engage in regular physical activity as a healthy lifestyle – which all participants in this study reported not doing. Such actions, as well as the reorganization of the workplace, should be encouraged, as they can prevent the development of health problems and improve physical work capacity scores.^23^

This study has some limitations. It is a cross-sectional study, in which we analyzed data collected for a limited period of time, without follow-up over time. Also, environmental comfort conditions were not considered, which can influence the results, making them less accurate. Future studies should have a longitudinal design and include the analysis of comfort conditions and their relationship to WMSDs.

## CONCLUSIONS

Our findings provide important information about musculoskeletal complaints and risk factors, showing that most of the interviewed workers, especially in the industrial sector, have painful complaints and perform their activities exposed to biomechanical risks within the Taylorist/Fordist model, with strong pressure to produce, excessive overtime, repetitive gestures, monotonous tasks, and unbearable speed requirements. Therefore, in view of the vulnerability of workers to WMSDs, employers should adequately assess occupational risks, including working posture analysis, to better adjust the workplace to individual biomechanical characteristics and increase the efficiency of movements within safe limits. In addition, rest breaks between activities and workplace exercise and stretching programs should be implemented, as well as the reduction of overtime, in order to prevent the development of these disorders and to improve workers’ performance.

## References

[1] Santana L, Sarquis L, Miranda F (2020). Riscos psicossociais e a saúde dos trabalhadores de saúde:
reflexões sobre a reforma trabalhista brasileira. Rev Bras Enferm.

[2] Hossain MD, Aftab A, Al Imam MH, Mahmud I, Chowdhury IA, Kabir RI (2018). Prevalence of work related musculoskeletal disorders (WMSDs) and ergonomic
risk assessment among readymade garment workers of Bangladesh: a cross sectional
study. PLoS One.

[3] Oranye NO, Bennett J (2018). Prevalence of work-related musculoskeletal and non-musculoskeletal injuries
in health care workers: the implications for work disability management. Ergonomics.

[4] Brendbekken R, Eriksen HR, Grasdal A, Harris A, Hagen EM, Tangen T (2017). Return to work in patients with chronic musculoskeletal pain:
multidisciplinary intervention versus brief intervention: a randomized clinical
trial. J Occup Rehabil.

[5] Abareshi F, Yarahmadi R, Solhi M, Farshad AA (2015). Educational intervention for reducing work-related musculoskeletal
disorders and promoting productivity. Int J Occup Saf Ergon.

[6] Brasil, Ministério da Saúde, Organização Pan-Americana da Saúde (2001). Doenças relacionadas ao trabalho: manual de procedimentos para os
serviços de saúde.

[7] Brasil, Ministério da Saúde (2019). Saúde Brasil 2018: uma análise da situação de
saúde e das doenças e agravos crônicos: desafios e
perspectivas.

[8] Moazzami Z, Dehdari T, Taghdisi MH, Soltanian A (2015). Effect of an ergonomics-based educational intervention based on
transtheoretical model in adopting correct body posture among operating room
nurses. Glob J Health Sci.

[9] Roquelaure Y, Bodin J, Descatha A, Petit A (2018). [Work-related musculoskeletal disorders]. Rev Prat.

[10] Lida I, Guimarães LBM (2016). Ergonomia: projeto e produção.

[11] Observatório de Segurança e Saúde no Trabalho (2018). Perfil dos afastamentos por agravo de 2012 a 2018 – Instituto Nacional do Seguro
Social (INSS).

[12] Viegas LRT, Almeida MMC (2016). Perfil epidemiológico dos casos de LER/DORT entre trabalhadores da
indústria no Brasil no período de 2007 a 2013. Rev Bras Saúde Ocup.

[13] Silva PLN, Monção MJD, Oliveira BLS, Cardoso TV, Soares LM, Costa ADA (2019). Distúrbio osteomuscular relacionado ao trabalho:
identificação dos fatores socioeconômicos e clínicos
autorreferidos por trabalhadores de saúde de uma instituição
hospitalar do município de Espinosa, Minas Gerais, Brasil. Rev Rede Cuid Saude.

[14] Silva Jr JS, Buzzoni GP, Morrone LC (2016). Queixas osteomusculares dos trabalhadores e condições
biomecânicas no trabalho em metalúrgica de alumínio. Rev Bras Med Trab.

[15] Abedini R, Choobineh A, Hasanzadeh J (2015). Patient manual handling risk assessment among hospital
nurses. Work.

[16] Melo IT, São-Pedro M (2012). Dor musculoesquelética em membros inferiores de pacientes obesos
antes e depois da cirurgia bariàtrica. ABCD Arq Bras Cir Dig.

[17] Cavuoto LA, Nussbaum MA (2014). The influences of obesity and age on functional performance during
intermittent upper extremity tasks. J Occup Environ Hyg.

[18] Maciel ACC, Fernandes MB, Medeiros LS (2006). Prevalência e fatores associados à sintomatologia dolorosa
entre profissionais da indústria têxtil. Rev Bras Epidemiol.

[19] Almeida CGSTG, Fernandes RCP (2017). Distúrbios musculoesqueléticos em extremidades superiores
distais entre homens e mulheres: resultados de estudo na
indústria. Rev Bras Saude Ocup.

[20] Milano JB, Siqueira MG (2015). Tratado de neurocirurgia.

[21] Brasil, Ministérios do Trabalho e Emprego (2018). Norma Regulamentadora No. 17 (NR-17): Ergonomia.

[22] Brasil, Presidência da República, Casa Civil, Subchefia para Assuntos Jurídicos (1977). Lei nº 6.514, de 22 de dezembro de 1977.

[23] Godinho MR, Ferreira AP, Greco RM, Teixeira LR, Teixeira MTB (2016). Capacidade para o trabalho e saúde dos vigilantes de uma
Universidade pública. Rev Latino-Am Enferm.

